# Evaluation of Protective Efficacy of Avicennia marina (Forssk.) Vierh Leaves against Complete Freund᾽s Adjuvant-induced Arthritis in Wistar 

**Published:** 2014

**Authors:** Mahdi Zamani Gandomani, Elaheh Forouzandeh Malati

**Affiliations:** a Department of Biology, Faculty of Science, Islamic Azad University of Hamedan Branch, Hamedan, Iran.; b Department of Marine Biology, Faculty of Oceanic and Marine Science, Khoramshahr Marine Science and Technology University, Iran.

**Keywords:** *Avicennia marina*, Complete freund᾽s adjuvant, Wistar rat

## Abstract

*Aviecennia marina *(Avicenniaceae) is an endemic plant that widely distributed in the Southern parts of Iran. This plant has been used as treatment of rheumatism arthritis among the inhabitants of Southern parts of Iran. The *Avicennia marina *hydroalcoholic extract was prepared and its protective efficacy was investigated using measurement of ankle diameter, total WBC and RBC count, ESR, and Pro-inflammatory cytokines levels in the complete Freund᾽s adjuvant (CFA)-induced arthritic rat. The increment in ESR and total WBC, reduction in RBC count and hemoglobin levels observed in the arthritic animals were also found to be significantly restored in HEA treated rats. *A. marina *at 400 mg/Kg significantly decreases the serum pro-inflammatory cytokines as well as normalizes ankle diameter of CFA rats. *A. marina *(400 mg/Kg) significantly normalizes changes observed in arthritic rats to near normal conditions, indicates that *A. marina *has promising protective efficacy against arthritic rats.

## Introduction

Rheumatoid arthritis (RA) is traditionally considered a chronic, inflammatory autoimmune disorder that causes the immune system to attack the joints, and afflicts generally 0.5-1.0% of the population around the world and commonly leads to significant disability and a consequent reduction in quality of life (1, 2, 3). Most commonly, small joints are affected, but larger joints can also be involved; the pattern of joint involvement can differ from patient to patient (4). Although the causes of RA remain unknown, several studies have revealed the key role of pro-inflammatory cytokine, such as tumor necrosis factor-α (TNF-α), interleukin-1β (IL-1β), and IL-6 in the pathogenesis of RA (5, 6, 7). TNF-α, IL-1β, and IL-6 are present abundantly in synovial fluid and joint tissues of RA patients (5). It has been suggested that these pro-inflammatory cytokines are produced through continuous activation of T cells and interaction of the activated T cells and monocytes/macrophages in RA (8). Moreover, anti-TNF, or anti-IL-1 and anti-LI-6 therapies have been reported to be affective in treatment of RA (9, 10). Currently, non-steroidal anti-inflammatory drugs (NSAIDs) supplemented with steroid hormone remains the major recommended strategy for its treatment (11, 12). While these drugs transiently suppress inflammation and ameliorate symptoms, they do not significantly improve the long-term disease outcome (13). Furthermore, long-term treatment with NSAIDs may result in serious side effects, such as gastrointestinal ulcergenicity and renal morbidity (1). Owing to these shortcomings, a more effective and safe therapeutic strategy is desired to treat RA. 

*Avicenna marina *(Forssk.) Vierh., (Avicenniaceae) is an aquatic native plant that grows abundantly in Southern parts of Iran, and it is popularly known as Harra. The bark, leaves, and fruits of *Aviecennia *are widely used in folk medicine for the cure and treatment of many ailments like skin disease, rheumatism arthritis, small pox, ulcers and fodder for livestock (14). It is a source of alcohols, amino acids, carbohydrates, fatty acids, hydrocarbons, inorganic salts, minerals, phytoalexins, carboxylic acids, steroids, tannins, triterpenes, vitamins, and iridoid glucosides (15-17). *A. marina *also exhibits anti-malarial and cytotoxic (18, 19), anti-cancer and anti-tumor (19) effects. No work has been carried out on the protective efficacy of this species against arthritis. Keeping in this view, the present study was undertaken to evaluate the protective effect of the hydroalcoholic extract of this species against CFA in rats. 

## Experimental

*Plant materials and extraction procedure*

The fresh leaves of *Avicennia marina *were collected from Nayband port, Boushehr province, Iran in April 2011. The plant materials were authenticated by Department of Botany (Faculty of Sciences, Isfahan University, Iran). A voucher specimen (No. 18068) was deposited at the Herbarium of Faculty of Science, Isfahan University, Isfahan, Iran.

For preparation of hydroalcoholic extract of *A. marina *(HEA), the leaves were washed, chopped, and air-dried under shade. Powder of leaves (100 g) was made and extracted by maceration in 400 mL of ethanol:water (7:3) at room temperature for 24 h. The extract was shaken and the extraction procedure was repeated once again under the same conditions with 250 mL of ethanol:water (7:3). Extracts were filtered through Wattman # 1 paper and evaporated to dryness in a rotary evaporator under reduced pressure. Polyphenolic fraction of the plant (200 g) was achieved in two steps, first, with ethanol:water (9:1) and then with ethanol:water (1:1). At each step sufficient solvent was added to make liquid slurry and mixture was left for 12 h. The two extracts were combined and evaporated to about 1/5 of the original volume. The resultant aqueous solution was cleared by extraction in a separating funnel with chloroform and then evaporated to dryness in a rotary evaporator under reduced pressure (20). Evaporation and solvent removal of hydroalcoholic extract and polyphenolic fraction gave semi-solid masses (yield 14 %). The dried material was stored under refrigeration at 4-8 ^o^C until its use.

*Preliminary phytochemical screening*

Preliminary phytochemical analysis of the extract was carried. The presence of alkaloids was tested with Dragendorff’s and Mayer’s reagents, flavonoids with HCl and Mg powder, phenols with ferric chloride, and steroids and terpenoids by Liebermann-Burchard reaction (21).

*Acute toxicity test*

According to OECD guide lines 423 female rats were selected and preceded. There were no signs of toxicity up to 4000 mg/Kg body weight. Based on the results obtained from this study, the dose for anti-inflammatory activity was fixed to be 200 mg/Kg body weight, and 400 mg/Kg body weight for dose dependent study.

*Animals*

A total of 30 healthy adult male Wistar rats (180-200 g) were used in this study. The rats were housed in wire cages under the controlled temperature (23 ± 3 ^o^C) and on a 12 h light-dark cycle (Lights on from 7:00) with food and water available ad libitum and acclimatized to animal house conditions. Under standardized conditions, rats were randomly subdivided into five groups each comprising of six animals normal rats. Group I served as normal controls. Group II, arthritic rats. Group III rats are arthritic ones that treated with oral ibuprofen (gavage) at a daily dose of 53 mg/Kg body weight (22). Group IV is comprised of arthritic rats were treated orally (gavage) with HEA (200 mg/Kg body weight). Group V arthritic rats were treated orally (gavage) with HEA (400 mg/Kg body weight). All experiments were carried out in accordance with local guidelines for the care of laboratory animals of Isfahan University of Medical Sciences, Isfahan, Iran.

*Arthritis induction*

Arthritis was induced by injecting subcutaneously 0.1 mL Complete Freund᾽s Adjuvant (CFA)(heat-killed *Mycobacterium tuberculosis *suspended in paraffin oil, purchased from Razi Institue, Karaj, Iran) into the sub plantar region of the right hind paw (23). Herbal and drug treatment was started from the initial day *i.e*. from the day of adjuvant induction (day 0), 30 minutes before adjuvant injection and continued till 30^th^ day. 

*Measurement of ankle diameter*

Ankle diameter in mm was determined using a caliper only twice: right before arthritis induction and at the end of the experiment (30 days after arthritis induction).

*Biochemical estimations*

On the day 30, Blood is withdrawn from each animal through retro-orbital vein puncture by anaesthetizing the animals with diethyl ether. The blood is collect into vials containing EDTA and the biochemical parameters like haemoglobin content, total WBC count, ESR and RBC analyzed (24). 

Levels of the pro-inflammatory cytokines TNF-α, IL-1β, and IL-6 in blood serum were measured on days 0, 17, and 30 using commercially available rat ELISA kits (Quantikine, R&D Systems *Inc*., Minneapolis, MN, USA) according to the manufacturer instructions.

*Statistical analysis*

The data were presented as mean ± standard error of the mean (SEM). Results were analyzed by one-way analysis of variance (ANOVA) followed by Tukey test multiple comparisons test with SPSS 19.00. p-values less than 0.01, and 0.05 were considered as indicative of significance.

## Results

*Effects of HEA on ankle diameter*

Our study showed that both doses of extract (200 and 400 mg/Kg), as well as ibuprofen, were effective in protecting the joints against deformity. The extract effectively inhibited redness, roughness, and erosion of the tested joints (Knee and ankle) (Table 1). Although the extract could not inhibit ankle swelling, it did inhibit deformity of its articular surfaces. In addition to, all the experimental groups showed a significant increment (p < 0.05) in the ankle diameter 30 days after CFA administration in comparison with the diameter before the adjuvant administration (Table 1). 

In this investigation, lesions developed at the paws and ankles of arthritic rats whereas in the extract treated rats the lesions developed over the paws only. In addition, functional disability manifested as difficulty in movement and dragging of the paw was observed in most of the arthritic rats, but not in the extract treated rats. The disability was accompanied with generalized hardness and darkness of the paws.

**Table 1:** Ankle diameter in mm (means ± SEM, n=6) of arthritic rats and rats treated with *A. marina *or ibuprofen.

**Day**	**Group I**	**Group II**	**Group III**	**Group IV**	**Group V**
**Zero**	5.0 ± 0.02	5.1 ± 0.03	4.8 ± 0.05	4.9 ± 0.01	4.9 ± 0.02
**Thirty**	6.6 ± 0.03*	6.9 ± 0.06*	6.6 ± 0.03*	6.7 ± 0.04*	6.4 ± 0.04*

* All means are significantly different (p < 0.05) from baseline (zero time) but there is no difference between treatment and control groups.

*Effects of HEA on biochemical estimations*

The level of ESR and total WBC count were significantly (p < 0.05) increased and RBC count and hemoglobin level were decreased in adjuvant induced rats when compared with normal group. Treatment with ibuprofen, and HEA showed significant (p < 0.05) decrease in the level of ESR and total WBC count and an increment in RBC count and hemoglobin level in comparison with adjuvant induced rats (Table 2).

**Figure 1:**
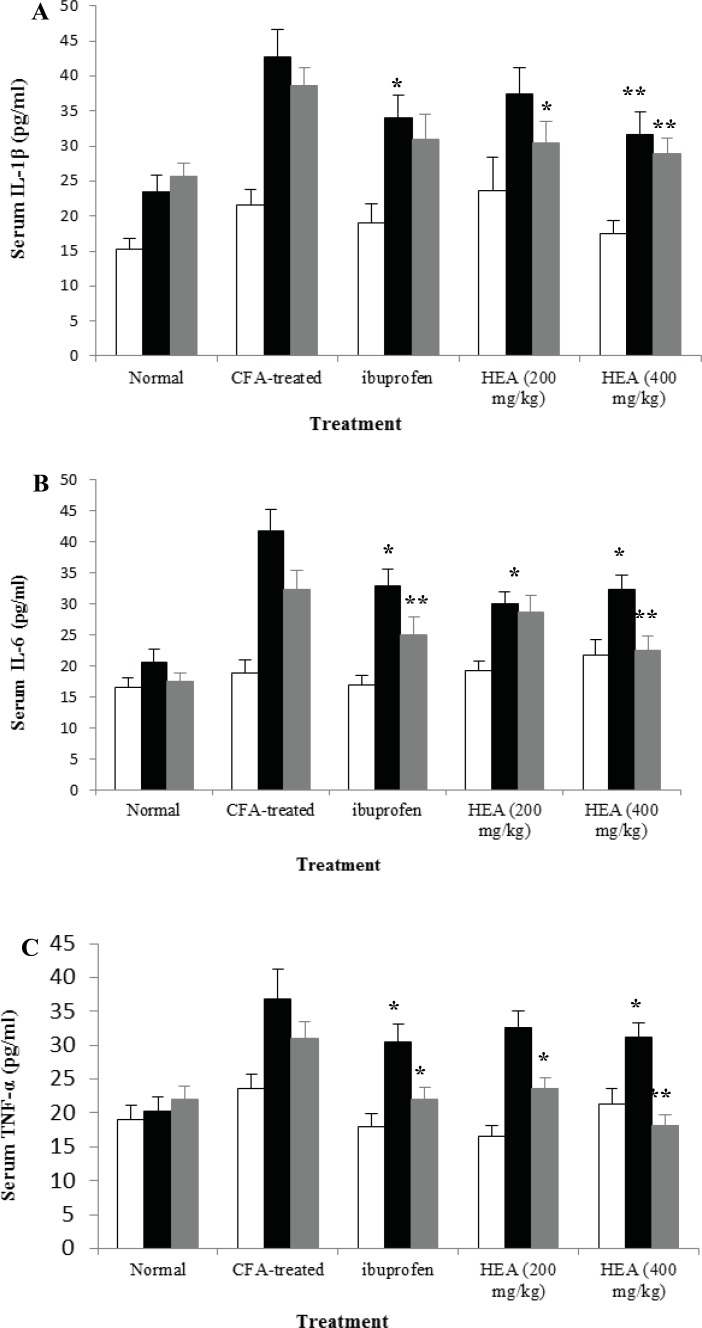
Effects of HEA on serum IL-1β (A), IL-6 (B), and TNF-α (C) levels of arthritic rats. Rats were daily orally treated with different doses of HEA, ibuprofen, beginning from day 0 (□), day 17 (■) or day 30 (▓) after arthritis induction until day 30. Data are expressed as means ± SEM (n=10). *p<0.05, and ** p<0.01 compared with arthritic rats.

Figure 1 shows levels of the pro-inflammatory cytokines IL-1β, IL-6, and TNF-α measured in blood samples collected on days 0, 17, and 30 of all groups. Consistent with the joint inflammation, IL-1β, IL-6, and TNF-α were significantly overproduced in the serum of adjuvant-induced arthritic rats with peak rises observed on day 17. The levels of these cytokines remain high despite slight declines with time; however, they were non-significant. Adjuvant-induced arthritic rats treated with *A. marina *extract had serum levels of these cytokines that were significantly and dose-dependently lower than arthritic rats. The reduction by *A. marina *extract of IL-1β, IL-6, and TNF-α is somewhat evident during their peak rise (day 17).

**Table 2:** Effects of HEA on the level of hemoglobin, ESR, total WBC count and RBC count in experimental groups

**ESR (mm/h)**	**RBC Count (x10** ^6^ **/mm** ^3^ **)**	**Total WBC Count (x10** ^3^ **/mm** ^3^ **)**	**Hemoglobin (g/dL)**	**Groups**
3 ± 0.17	3.6 ± 0.12	6.8 ± 0.66	11.5 ± 0.24	Group I
11 ± 0.33^a^	2.52 ± 0.2^a^	15.2 ± 0.7^a^	7.6 ± 0.87 ^a^	Group II
5 ± 0.12^b^	3.34 ± 0.03	10.5 ± 0.085^b^	8.9 ± 0.45	Group III
6 ± 0.13^b^	3.71 ± 0.03	10.2 ± 0.91^a,b^	9.87 ± 0.5	Group IV
4 ± 0.13^b^	3.00 ± 0.04	10.9 ± 0.9^a,b^	9.1 ± 0.21	Group V

Data are expressed as means ± SEM (n = 6). a-Statistical difference with control group at p < 0.05. b-Statistical difference of group II with group III, IV, and group V at p < 0.05.

## Discussion

In the present study, rats were selected to induce arthritis because rats develop a chronic swelling in multiple joints with influence of inflammatory cells, erosion of joint cartilage and bone destruction. It has close similarities to human rheumatoid arthritis (25, 26). Chronic inflammation involves the release of number of mediators like cytokines (IL-1β, IL-6, and TNF-α), GM-CSF, interferon᾽s, and PGDF. These mediators are responsible for the pain, destruction of bone and cartilage that can lead to severe disability (27).

In the present investigation the arthritis rats showed a soft tissue swelling that was noticeable around the ankle joints during the acute phase of arthritis and was due to be edema of periarticular tissues such as ligaments and joint capsules. The swelling has been found to be increasing in the initial phase of inflammation and then becomes constant in 2 week. These changes in ankle diameter probably have been found to be associated with an increase in granulocyte and monocytes (28).

In the present study, lesions were observed at the paws and ankles of arthritic rats, but not in treated rats. It seemed that the process involved in the development of lesion does not appear to be infectious, but rather a generalized immunological response to the constituents of the tubercle bacilli (29). 

The arthritic rats exhibited a reduced RBC count, reduced hemoglobin level and an increased ESR. All these indicated the anemic condition which is a common diagnostic feature in patients with chronic arthritis (30). In arthritic conditions, there is an increment in WBC count due to release of IL-1β inflammatory response. IL-1β increases the production of both granulocyte and macrophages colony stimulating factor (27). The treatment with ibuprofen or HEA improved the RBC count, hemoglobin level and ESR to a near normal level indicating the significant recovery from the anemic condition. The migration of leukocytes into inflamed area is also significantly suppressed by HEA as seen from the significant decrease in total WBC count.

Although the exact molecular mechanisms responsible for cartilage and bone destruction have not been elucidated, studies have shown that pro-inflammatory cytokines TNF-α, IL-1β, and IL-6 play a critical role in the pathological process of arthritis (5, 6). It has been long speculated that RA could be triggered by a T-cell response to infectious agents. Through cell-cell contact and different cytokines, these stimulated T-cells activate monocytes, macrophages, and synovial fibroblasts. The latter then overproduce pro-inflammatory cytokines, mainly TNF-α, IL-1β, and IL-6 (31). In addition, TNF-α and IL-1 also induce receptor activator of nuclear factor-kB (RANK) on macrophages which differentiate into osteoclasts that resorb and destroy bone (31). These soluble molecules, once engaged to their receptors, trigger various signal transduction cascades that lead to the activation of transcription factors and the subsequent induction of genes whose products such as matrix metalloproteinase (MMPs) mediate tissue degradation (32). TNF-α is mainly involved in the perpetuation of the inflammatory cascades in autoimmune diseases, which affect connective tissues where the connective tissues become hypercontracted due to inflammation (33). In the present study, *A. marina *extract lowered elevated cytokines. This indicates that the extract might lower autoantibody production by a mechanism that is unlikely to be dependent only on the reduction of IL-1β, IL-6, and TNF-α levels and may be related to an additional effect on B cell function or due to an effect on other Th1 or Th2 cell cytokines or chemokines that was not measured. 

## Conclusion

In conclusion, *A. marina *(400 mg/Kg) significantly normalizes changes observed in arthritic rats to near normal conditions, as it inhibited the CFA-induced skin lesions and articular deformity and indicates that *A. marina *has promising protective efficacy against arthritic rats. So, the hydroalcoholic extract of *Avicennia marina *is safe at high dose acutely. These results support the use of *A. marina *as a herbal medicine for treatment of inflammatory disorders and rheumatoid arthritis.
